# Human perception of electrical stimulation on the surface of somatosensory cortex

**DOI:** 10.1371/journal.pone.0176020

**Published:** 2017-05-10

**Authors:** Shivayogi V. Hiremath, Elizabeth C. Tyler-Kabara, Jesse J. Wheeler, Daniel W. Moran, Robert A. Gaunt, Jennifer L. Collinger, Stephen T. Foldes, Douglas J. Weber, Weidong Chen, Michael L. Boninger, Wei Wang

**Affiliations:** 1 Department of Physical Medicine and Rehabilitation, University of Pittsburgh, Pittsburgh, Pennsylvania, United States of America; 2 Center for the Neural Basis of Cognition, Pittsburgh, Pennsylvania, United States of America; 3 Department of Physical Therapy, Temple University, Philadelphia, Pennsylvania, United States of America; 4 Department of Bioengineering, University of Pittsburgh, Pittsburgh, Pennsylvania, United States of America; 5 Department of Neurological Surgery, University of Pittsburgh, Pittsburgh, Pennsylvania, United States of America; 6 McGowan Institute for Regenerative Medicine, University of Pittsburgh, Pittsburgh, Pennsylvania, United States of America; 7 Department of Biomedical Engineering, Washington University in St. Louis, St. Louis, Missouri, United States of America; 8 Department of Veterans Affairs Medical Center, Pittsburgh, Pennsylvania, United States of America; 9 Barrow Neurological Institute at Phoenix Children’s Hospital, Phoenix, Arizona, United States of America; 10 Qiushi Academy for Advanced Studies, Zhejiang University, Hangzhou, Zhejiang, China; 11 Clinical and Translational Science Institute, University of Pittsburgh, Pittsburgh, Pennsylvania, United States of America; 12 Mallinckrodt Institute of Radiology, Washington University School of Medicine and Barnes-Jewish Hospital, St. Louis, Missouri, United States of America; University of California Los Angeles, UNITED STATES

## Abstract

Recent advancement in electrocorticography (ECoG)-based brain-computer interface technology has sparked a new interest in providing somatosensory feedback using ECoG electrodes, i.e., cortical surface electrodes. We conducted a 28-day study of cortical surface stimulation in an individual with arm paralysis due to brachial plexus injury to examine the sensation produced by electrical stimulation of the somatosensory cortex. A high-density ECoG grid was implanted over the somatosensory and motor cortices. Stimulation through cortical surface electrodes over the somatosensory cortex successfully elicited arm and hand sensations in our participant with chronic paralysis. There were three key findings. First, the intensity of perceived sensation increased monotonically with both pulse amplitude and pulse frequency. Second, changing pulse width changed the type of sensation based on qualitative description provided by the human participant. Third, the participant could distinguish between stimulation applied to two neighboring cortical surface electrodes, 4.5 mm center-to-center distance, for three out of seven electrode pairs tested. Taken together, we found that it was possible to modulate sensation intensity, sensation type, and evoke sensations across a range of locations from the fingers to the upper arm using different stimulation electrodes even in an individual with chronic impairment of somatosensory function. These three features are essential to provide effective somatosensory feedback for neuroprosthetic applications.

## Introduction

Cortical surface stimulation through electrocorticography-based (ECoG) electrodes has been used by clinicians since the 1930s to identify various sensory and motor areas for presurgical brain mapping [1, 2]. Given recent advancements in brain-computer interface studies using ECoG electrodes [3–6], there is an increased interest in providing somatosensory feedback using cortical surface electrodes. This leads to the question: is it possible to change the intensity, type, and location (body part) of artificially elicited sensation in a controlled fashion by manipulating parameters of cortical surface stimulation? A number of animal studies [7–13] and a recent human study [14] demonstrated the feasibility of somatosensory feedback using intracortical microstimualtion. However, only a limited number of studies have evaluated delivery of somatosensory information to people using cortical surface stimulation [15–17]. A review paper by Borchers et al. indicated that cortical surface stimulation could evoke complex bioelectrical and neurophysiological effects in a large volume of the brain with unpredictable behavioral responses [15]. Their paper further suggested that stimulating the same cortical site could result in drastically different behavioral effects ranging from evocation to inhibition of a response. Hence, it is imperative to systematically evaluate the sensation produced by cortical surface stimulation at the somatosensory cortex. Wheeler et al. demonstrated that non-human primates could detect electrical stimulation of the somatosensory cortex through ECoG electrodes in their bi-directional brain-computer interface study [18]. Johnson et al. conducted a study in two individuals who were undergoing clinical ECoG monitoring for surgical treatment of epilepsy [16]. Their participants could discriminate between high and low intensity stimulation 84% of the time, and the same group further showed the feasibility of human subjects modulating their grasping movement based on feedback from cortical surface stimulation [17]. Building upon previous studies, we conducted a 28-day cortical surface stimulation study in an individual with left arm paralysis from brachial plexus injury. The current study uniquely combines the following features: 1) direct report of elicited sensation by a human subject; 2) extended study time as compared to studies in patients undergoing clinical brain mapping; 3) high-density cortical surface electrode grid (60 electrodes in 4 cm × 4 cm grid) implanted in the targeted somatosensory area; and 4) a translational study in a participant who is among the target clinical population of individuals with somatosensory deficit. The goal of the current study is to systematically characterize the change in intensity, type, and location (body part) of the evoked sensation as we change stimulus train duration, time gap between two stimulus trains, pulse amplitude, pulse frequency, pulse width, and cortical location of the stimulating electrode.

## Materials and methods

### Study participant

The study was approved by the Institutional Review Board at the University of Pittsburgh and registered at clinicaltrials.gov (NCT01393444) [6]. Written informed consent was obtained from the participant before study initiation. The study was conducted as part of an ongoing study that involved both stimulation and recording. Only the stimulation results are reported here.

The participant was a 24-year-old right-handed male with left brachial plexus injury and left cervical (C6-8) nerve root avulsion due to an accident three years prior to this study, and he had no volitional left arm or hand movement. The participant underwent intercostal nerve transfer surgery fifteen months post brachial plexus injury at another institution. While we were unable to obtain the operative report that provided details of the nerve transfer surgery, the participant reported that he recovered partial sensation in the triceps region of the left upper arm but without any recovery of motor function. Clinical electromyography (EMG) and nerve conduction studies performed as a part of the participant’s clinical care two years post-injury reported a severe global left brachial plexus lesion, affecting all nerve trunks, and an absence of sensory and motor responses of the left medial, ulnar and radial nerves. The participant had an implanted spinal cord stimulator (cervical dorsal column) for pain control, which was turned off during all testing sessions. Muscle testing was performed by a physician, who indicated no visible or palpable contraction for shoulder flexors and extensors, elbow flexors and extensors, wrist extensors, and finger flexors and abductors for the left arm [19]. Dermatome testing indicated no sensation for left cervical level C4-C8 and altered sensation for C2-C3 and T1-T2.

### High-density ECoG grid and stimulation instrumentation

**Fig 1:**
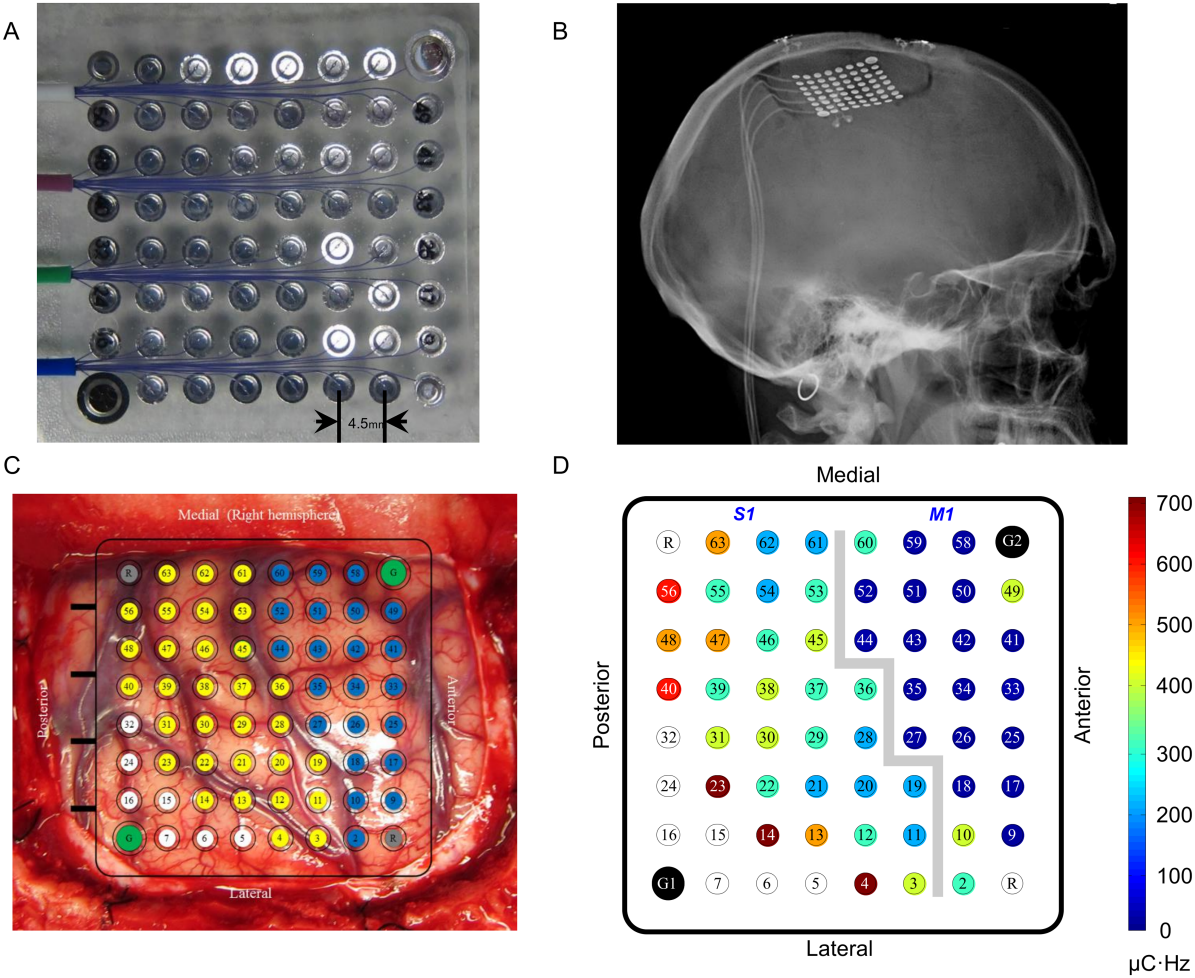
ECoG grid and activation thresholds. ***A***: Photograph of the high-density ECoG grid used in this study. The camera is looking at the back of the grid, the non-brain facing surface. The grid was composed of a silicone sheet (4 cm × 4 cm in size, 1 mm thick) with 64 platinum electrodes. The electrodes were 2 mm in diameter and 4.5 mm apart (center-to-center), except for the two ground electrodes that were 3 mm in diameter and 5 mm from the neighboring electrodes (center-to-center). All electrodes were connected to platinum lead wires that formed four 60-cm long leads. ***B***: Post-operative skull radiograph showing the ECoG grid. ***C***: Grid location on brain surface. The blue electrodes are above the motor cortex (M1). The yellow electrodes are above the somatosensory cortex (S1). The green and grey electrodes are dura-facing ground and reference electrodes, respectively. The white electrodes did not generate a response to any cortical stimulation attempted within the safety limits of our study (less than 57 μC/cm^2^ per phase). ***D***: Activation thresholds. G1 and G2 (black electrodes) were the return electrodes for Electrodes 1–32 and Electrodes 33–64, respectively. The threshold values are presented in terms of charge exchange per second (μC•Hz) required to elicit a response. The gray line separates the electrodes above the motor and somatosensory cortices. The stimulation pulse amplitude, width, and frequency ranged from 1 to 7 mA, 50 to 400 μs, and 50 to 500 Hz, respectively. The color bar represents the range of the charge exchange.

We used a custom-designed high-density ECoG grid (PMT Corp, Chanhassen, MN USA) with 64 platinum disc electrodes (Fig 1A). The grid was composed of a silicone sheet (4 cm × 4 cm in size, 1 mm thick) with 60 electrodes facing the brain, two ground electrodes facing the dura mater, and two reference electrodes also facing the dura mater. The electrodes were 2 mm in diameter and 4.5 mm apart (center-to-center), except for the two ground electrodes that were 3 mm in diameter and 5 mm from the neighboring electrodes (center-to-center). All electrodes were connected to platinum lead wires that formed four 60-cm long leads with a total of 64 standard ring connectors. A CereStim^™^ R96^™^ macro-neurostimulator (Blackrock Microsystems LLC, Salt Lake City, UT USA) was used to provide monopolar, charge-balanced, current-controlled, biphasic square pulse cortical surface stimulation to the participant. Electrodes 1 to 32 used Electrode G1 (next to Electrode 7) as the return electrode, and Electrodes 33–63 used Electrode G2 (next to Electrode 58) as the return electrode (Fig 1D). StimManager PC software (Blackrock Microsystems LLC, Salt Lake City, UT USA) was used to program and trigger stimulation.

### Presurgical brain mapping and surgical procedure

Functional mapping of the primary somatosensory and motor cortices was performed to guide craniotomy and electrode placement prior to the implantation surgery. Magnetic resonance imaging (MRI) was not performed for this study because the participant’s spinal cord stimulator was not MRI-compatible. Instead, magnetoencephalography (MEG) data collected during sensory and motor tasks were used to identify cortical areas contra-lateral to the paralyzed left arm and hand [14, 20]. To map the location of the right somatosensory cortex during MEG data collection, electrical stimulation was delivered to the participant’s triceps region of the left upper arm, where there was residual sensation. Furthermore, MEG data were collected while the participant’s left thumb, little finger, and palm were manually brushed in synchrony with a video. Though the participant could not feel the brushing, he was asked to imagine the sensation as demonstrated on his right hand (the intact side). This paradigm was used to engage the mirror neuron system and the somatosensory areas [6, 14, 20]. The right motor cortex was mapped using various movements, including shoulder shrugging, elbow flexion/extension, hand grasping, and individual finger movements. The participant could not perform these movements except for shoulder shrugging, but was asked to attempt the movements.

One high-density ECoG grid was implanted subdurally above the hand and arm areas of the right pre- and post-central gyri (Fig 1), and electrode impedance was tested using XLTEK^®^ Protektor clinical neurophysiological monitoring system (Natus Medical Incorporated, Pleasanton, CA). The four leads of the ECoG grid were tunneled subcutaneously to the right chest and exited the skin inferior to the clavicle. A sterile dressing covered the exit site, and the leads were physically connected to the neural stimulation system during testing. The participant stayed in the hospital for two nights after the implantation surgery, and testing commenced on Post-operative Day 3. The grid was explanted on Day 28 per our IRB protocol.

### Cortical surface stimulation sessions

Seventeen sessions of sensory stimulation were conducted over 22 testing days, and each session typically lasted from one to three hours. We stayed under the safety limits of charge density proposed by previous studies (less than 57 μC/cm^2^ per phase) [21, 22]. There were no seizures or other complications from electrical stimulation conducted in this study. The cortical stimulation study progressed through three phases: 1) cortical mapping, 2) stimulation detection, and 3) stimulation discrimination and quantification. Each phase generated results that informed the next phase. For clarity of discussion, several key terms were defined for this study as listed in Table 1.

**Table 1:** Terminology and definition.

Terminology	Definition
Charge exchanged per second (μC•Hz)	Pulse amplitude × Pulse width × Pulse frequency. This value allowed us to compare the activation threshold across different electrodes and stimulus trains of different pulse amplitudes, widths, and frequencies.
Activation threshold (μC•Hz)	Minimal charge exchanged per second required to elicit a sensory or motor response for stimulation through a specific electrode. This was established in Phase 1 (cortical mapping).
Detection amplitude (mA)	Minimal pulse amplitude to elicit a sensory response for stimulation through a specific electrode. This was established in Phase 2 (stimulation detection).
Time gap (s)	Time gap between a pair of stimulus trains.
Sensation field	Specific part of the body where the participant felt a sensation during cortical stimulation through a specific electrode.
Trial	During each trial, either a single stimulus train was delivered or two stimulus trains were delivered sequentially separated by a time gap.
Block	Each block had multiple trials tested in a pseudo-random order. Each block focused on a single electrode (except for the spatial discrimination test), and only one stimulation parameter was varied across trials within a block unless specified otherwise.

**Fig 2:**
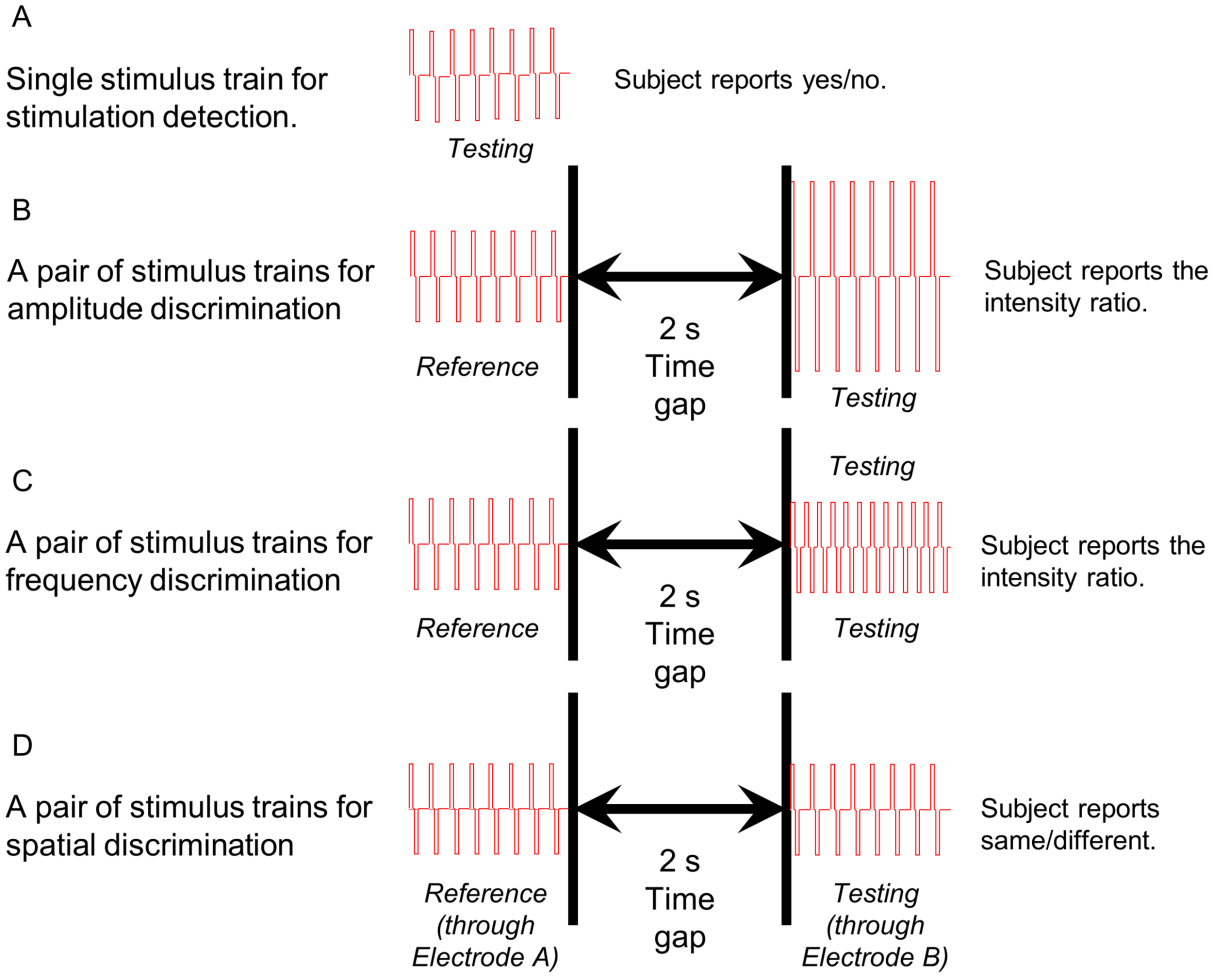
Stimulation paradigms. A stimulus train is a group of bi-phasic pulses drawn in red. This figure is not drawn to actual time scale.

Fig 2 and Table 2 show the stimulation paradigms and their corresponding parameters, respectively.

**Table 2:** Stimulation paradigms and their corresponding parameters. Detection amplitude was specific to each electrode that is tested, and it was established in Phase 2 (Stimulation detection). Detection amplitude was also established for 200 and 400 μs pulse widths for a subset of electrodes following the same style as Phase 2 (Stimulation detection). Δ50 Hz and Δ10 Hz mean 50Hz and 10Hz increments, respectively.

Test name	Pulse amplitude (mA)	Pulse width (μs)	Pulse frequency (Hz)	Train duration (s)	Electrodes and repetitions per electrodes	Time gap Btw two trains (sec)
**Min amplitude (detection amplitude)**	Varying	400	500	1	11 electrodes and 10 repetitions	N/A
**Min stimulus train duration**	Detection amplitude	400	500	Varying	6 electrodes and 10 repetitions	N/A
**Min time gap**	Detection amplitude	400	500	1	2 electrodes and 10 repetitions per amplitude	Varying from 0 to 6
**Amplitude discrimination and quantification**	Varying	400	500	0.5	4 electrodes and 10 repetitions per amplitude	2
**Frequency discrimination and quantification**	Detection amplitude	400	Varying	0.5	4 electrodes and 3 repetitions for Δ50 Hz, 10 repetitions for Δ10 Hz	2
**Sensation type discrimination**	Detection amplitude for 200 and 400 μs pulse widths	200 or 400	500	0.5	8 electrodes and 10 repetitions for each pulse width	N/A
**Spatial discrimination**	Detection amplitude	400	500	1	7 electrode pairs and 10 repetitions per pair	2

### Phase 1: Cortical mapping

The study began by identifying electrodes overlying the somatosensory and motor cortices using cortical surface stimulation under direct supervision of a neurosurgeon. The initial stimulation parameters were based on previous humans studies, our clinical protocols for pre- and intra-operative cortical mapping, and a study conducted in non-human primates [16, 18]. Two investigators observed the participant for any overt movement elicited by electrical stimulation. The participant was instructed to inform investigators if he felt sensation at any body part. The participant also marked sensation fields on schematic hand and arm pictures. The cortical mapping session also established the activation thresholds to elicit any motor or sensory responses.

### Phase 2: Stimulation detection

We used a two-alternative forced choice task to identify the minimum pulse amplitude (detection amplitude) and stimulation duration (Table 2). We required the participant to detect the stimulation with an accuracy of 75% or higher. To detect minimum pulse amplitude a 1-s stimulus train was delivered, and the participant had to report either ‘yes’ or ‘no’ to indicate whether he felt the stimulation. Multiple pulse amplitudes with resolution of 0.1 mA mixed with sham stimulation (no stimulation) were tested in a pseudo-random order in each block. The detection amplitude was used in subsequent stimulation testing. To identify the minimal duration required a set of stimulation durations ranging from 0.1 to 1 s with resolution of 0.1 s were tested in each block. To identify the minimal time gap required for the participant to detect sequential delivery of two stimulus trains instead of one, we used the same two-alternative forced choice task. A set of time gaps ranging from 0 to 6 s with resolution of 1 s was tested in each block.

### Phase 3: Stimulation discrimination and quantification

Each trial of the amplitude and frequency discrimination tasks consisted of a reference stimulus train followed by a test stimulus train (Fig 2 and Table 2). The reference amplitude was set to the detection amplitude established in Phase 2, and various amplitudes were tested in each block. The reference frequency was first set to 500 Hz with the testing frequency varying between 50 and 500 Hz (50 Hz resolution) in each block. The reference frequency was then set to 200 Hz with the testing frequency varying between 100 to 200 Hz (10 Hz resolution) in each block. The participant reported an intensity ratio between the testing and reference stimulus trains. For example, 0% indicated no testing stimulus train was felt, and 200% indicated the testing stimulus train was twice as strong as the reference stimulus train.

For sensation type discrimination, each trial used a single stimulus train. We tested two pulse widths, 200 μs and 400 μs, and we set the pulse amplitude to the detection amplitude specifically identified for each pulse width. Each block tested two different combinations of pulse width and pulse amplitude in a pseudorandom order. The participant indicated which one of the two types of sensation (electrical buzz vs. tingling) he felt. For spatial discrimination, each trial delivered a pair of stimulus trains sequentially, reference and testing stimulus trains. The participant indicated whether the sensations were the same or different. Each block tested two electrodes. The reference stimulus train was always delivered through the first electrode, and the testing stimulus train was delivered through either the first or second electrode determined in a pseudorandom fashion. The participant also marked the sensation fields on schematic hand and arm pictures.

## Results

### Cortical mapping

**Fig 3:**
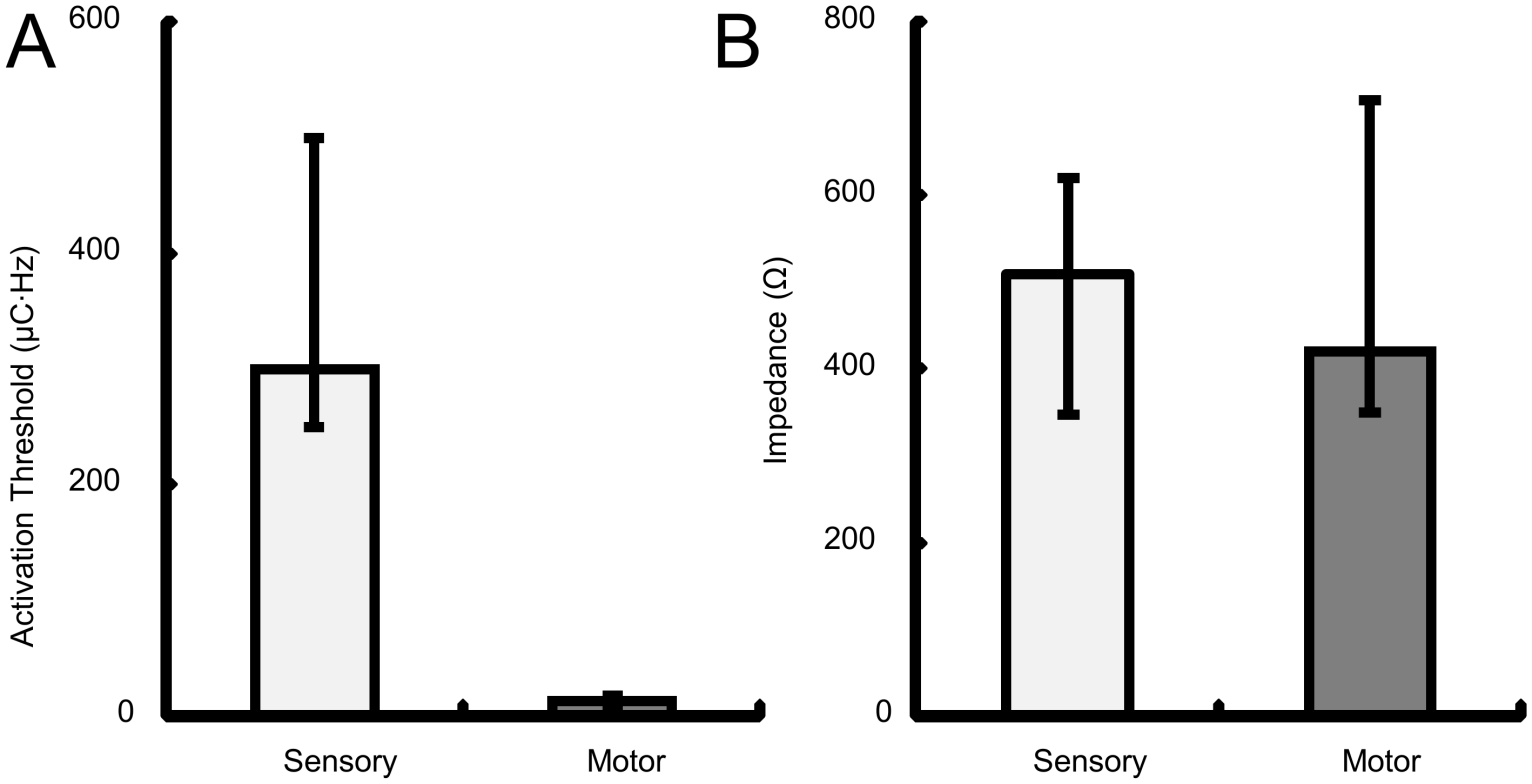
Motor and somatosensory cortical electrode activation thresholds and electrode impedances. ***A***: Median and interquartile range of activation thresholds for electrodes above the somatosensory cortex (light gray) and those above the motor cortex (dark gray). Activation thresholds were significantly different between the somatosensory and motor cortical electrodes (Wilcoxon rank sum test, p<0.05). ***B***: Median and interquartile range of impedances for electrodes above the somatosensory cortex (light gray) and those above the motor cortex (dark gray). Electrode impedances were tested at the end of the implantation surgery using the clinical XLTEK^®^ system (non-programmable default testing signal frequency of 1000 Hz). There was no significant difference in electrode impedance between the somatosensory and motor cortical electrodes (Wilcoxon rank sum test, p = 0.66).

**Fig 4:**
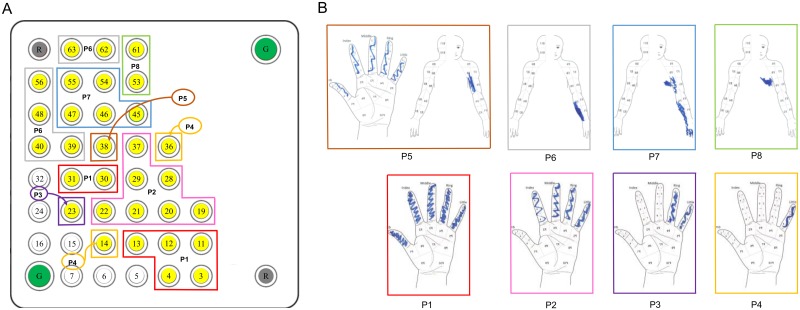
Sensation fields perceived by the participant. ***A***: Electrode groups that correspond to the eight patterns of evoked sensations indicated by the participant. The white electrodes had no response to cortical stimulation. ***B***: The eight patterns of activity, corresponding to various sensation fields, perceived by the participant during stimulation of the sensory electrodes. The sketches were made by the participant using his right hand on a template.

Fig 1 shows the estimated location of the 64-electrode ECoG grid. Stimulation through electrodes above the motor cortex generally elicited shoulder movement or facial muscle twitching. Stimulation through the electrodes above the somatosensory cortex evoked various sensations, including electrical buzz, tingling, vibration, sensation of arm movement (Electrodes 55 and 61), and vertigo (Electrodes 53 and 62 at pulse amplitude of 6–7 mA, much higher than their detection amplitudes). The activation threshold for electrodes above the motor cortex was significantly lower than the activation threshold for electrodes above the somatosensory cortex (Wilcoxon rank sum test, p<0.05) (Figs 1D and 3A; Table 3). This was not simply caused by a systematic difference in electrode-tissue interface properties, e.g., effective contact area, between the motor and sensory cortical electrodes, as there was no significant difference in impedance between sensory and motor cortical electrodes (Wilcoxon rank sum test, p = 0.66) (Fig 3). The rest of the study focused on the electrodes above the somatosensory cortex (yellow electrodes in Fig 1C). Eight patterns of sensation fields were perceived by the participant during stimulation through these electrodes (Fig 4). The sensation field perceived by the participant shifted from hand to arm as the location of the stimulation electrode changed from the lateral to the medial portion of the ECoG grid, consistent with the somatotopy of the somatosensory cortex [2, 23].

**Table 3:** Electrodes and the corresponding activation thresholds (stimulation parameters needed to elicit a motor or sensory response).

Motor Electrode	Amplitude (mA)	Pulse width (μs)	Frequency (Hz)	Activation Threshold (μC∙Hz)	Sensory Electrode	Amplitude (mA)	Pulse width (μs)	Frequency (Hz)	Activation Threshold (μC∙Hz)
***2***	3	200	500	300	***3***	2	400	500	400
***9***	4	50	50	10	***4***	3.5	400	500	700
***10***	4	200	500	400	***11***	1	400	500	200
***17***	5	50	50	12.5	***12***	1.5	400	500	300
***18***	5	50	50	12.5	***13***	2.5	400	500	500
***25***	3	50	50	7.5	***14***	3.5	400	500	700
***26***	3	50	50	7.5	***19***	1	400	500	200
***27***	5	50	50	12.5	***20***	1	400	500	200
***33***	2	200	50	20	***21***	1	400	500	200
***34***	5	50	50	12.5	***22***	1.5	400	500	300
***35***	5	50	50	12.5	***23***	3.5	400	500	700
***41***	4	50	50	10	***28***	1	400	500	200
***42***	4	50	50	10	***29***	1.5	400	500	300
***43***	4	50	50	10	***30***	2	400	500	400
***44***	7	50	50	17.5	***31***	2	400	500	400
***49***	4	200	500	400	***36***	1.5	400	500	300
***50***	4	50	50	10	***37***	1.5	400	500	300
***51***	4	50	50	10	***38***	2	400	500	400
***52***	6	50	50	15	***39***	1.5	400	500	300
***58***	5	50	50	12.5	***40***	3	400	500	600
***59***	6	50	50	15	***45***	2	400	500	400
***60***	3	200	500	300	***46***	1.5	400	500	300
					***47***	2.5	400	500	500
					***48***	2.5	400	500	500
					***53***	1.5	400	500	300
					***54***	1	400	500	200
					***55***	1.5	400	500	300
					***56***	6	200	500	600
					***61***	1	400	500	200
					***62***	1	400	500	200
					***63***	2.5	400	500	500

### Stimulation detection

A stimulus train needed to last at least 0.2 s long for it to be detected by the participant more than 75% of the time. We set the stimulation duration to 0.5 s (detected 100% of the time) for the rest of the study. The time gap between two stimulus trains needed to be at least 1 s for the participant to recognize that there were two separate stimulus trains 75% of the time. We set the time gap between a pair of stimulus trains (reference and testing) to 2 s for the rest of the study.

### Stimulation discrimination and quantification

#### Sensation intensity as a function of pulse amplitude

**Fig 5:**
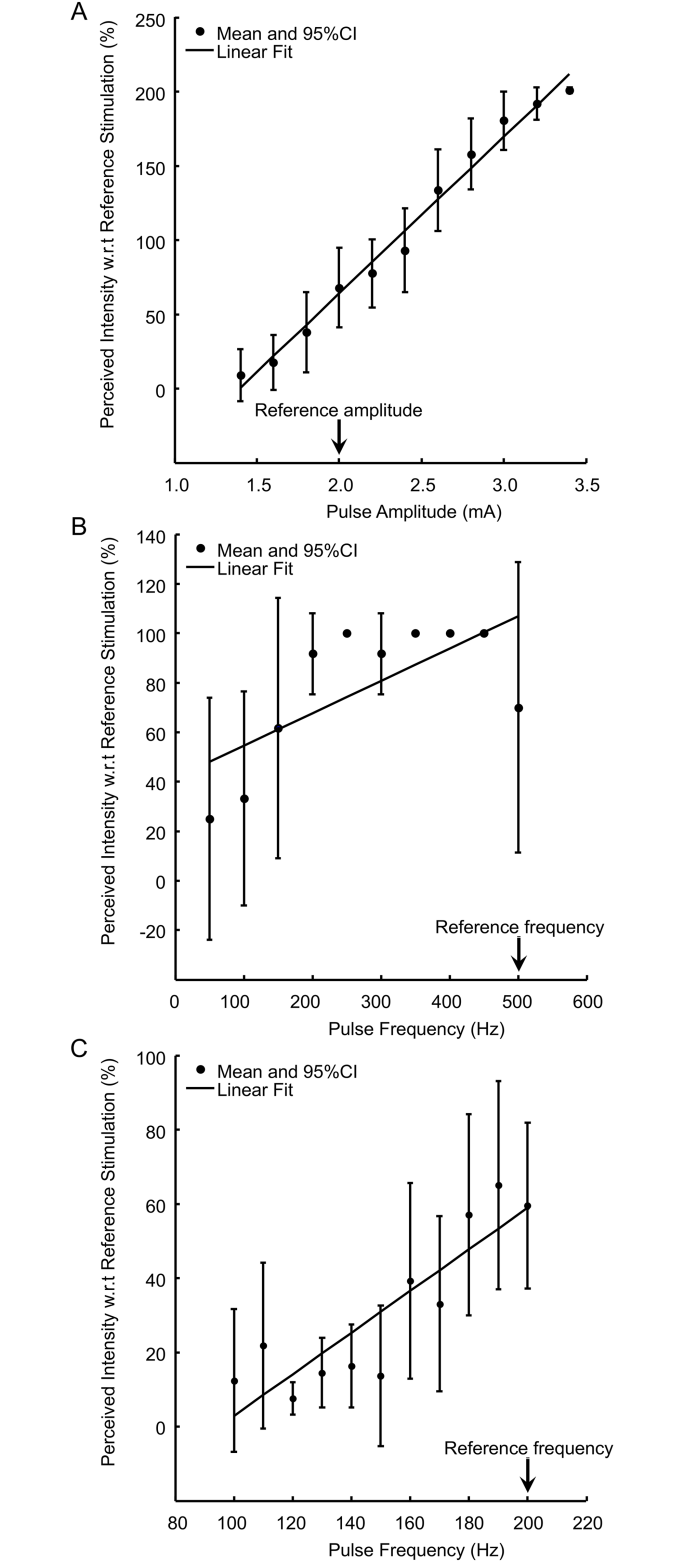
Perceived intensity as a function of stimulation pulse amplitude and frequency. The figures indicate the percentage of perceived intensity (y-axis) for a second stimulus train with respect to a preceding reference stimulus train. All trials consisted of two stimulus trains separated by a 2 s time gap. The x-axis indicates the pulse amplitude or frequency. The reference pulse amplitude and frequency are marked by black arrows. The black dots and error bars represent mean and 95% confidence interval, and the linear fit was drawn as a black line. ***A***: The figure corresponds to 10 repetitions of stimulations at Electrode 38 (Linear fit: R^2^ = 0.99, p< 0.05). A similar monotonic pattern was observed with three other electrodes (3, 11, and 20). ***B***: The figure corresponds to 3 repetitions of stimulations at Electrode 38 (Linear fit: R^2^ = 0.48, p< 0.05). A similar pattern was observed with three other electrodes (3, 20, and 46). ***C***: Perceived intensity reported by the participant for a smaller frequency range (100 to 200 Hz) compared to ***B***. The figure corresponds to 10 repetitions of stimulations at Electrode 38 (Linear fit: R^2^ = 0.71, p< 0.05). A similar pattern was observed with three other electrodes (3, 20, and 46).

We found that there was a graded, linear relationship between pulse amplitude and the perceived intensity for three of the four electrodes tested (R^2^ values were 0.70, 0.95, 0.99 for Electrodes 3, 11, and 38, respectively, for linear fits; p<0.05) (Fig 5A). The perceived intensity was not significantly related to pulse amplitude for Electrode 20. A secondary finding was that the perceived intensity for the second stimulus train was approximately 70% as strong as the first stimulus train when two identical stimulus trains were delivered with a 2 s time gap (Fig 5A).

#### Sensation intensity as a function of pulse frequency

We observed that the participant’s perceived intensity increased when the pulse frequency was increased between 100–200 Hz and then plateaued (Fig 5B). When we further tested pulse frequencies between 100 and 200 Hz, we found that 150–200 Hz was the frequency range where change in pulse frequency led to most significant change in perceived intensity. There was a linear relationship between pulse frequency and the perceived intensity for all four electrodes we tested (R^2^ values were 0.88, 0.87, 0.72, and 0.63 for Electrodes 3, 20, 38, and 46, respectively; p<0.05 for all electrodes) (Fig 5C).

#### Sensation type discrimination

**Fig 6:**
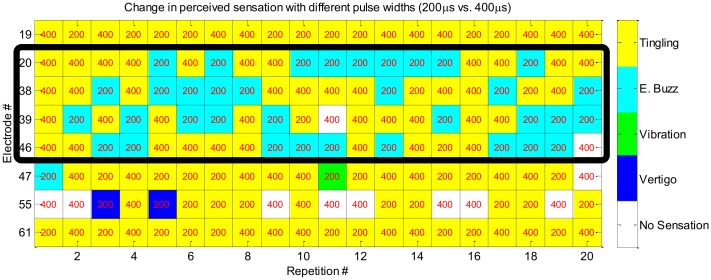
Perceived sensation for two types of stimulus trains. The two types of stimulus trains were 0.5 s in duration and had pulse widths of 200 μs and 400 μs. The participant reported two distinct sensations (tingling and electrical buzz) for four of the eight electrodes (20: 100%. 38: 90%. 39: 90%. 46: 90%) when the stimulus train pulse width was changed from 400 μs (10 repetitions) to 200 μs (10 repetitions). The sensation types of tingling, electrical buzz (E. Buzz), vibration, and vertigo are indicated by yellow, cyan, green, and blue colored tiles, respectively. The white colored tiles indicate no perceived sensation.

During the cortical mapping phase, we found that the sensation type for some electrodes changed when the pulse width changed from 200 μs to 400 μs. This led us to assess whether there was a relationship between sensation type and pulse width. The participant reliably distinguished two types of sensation, electrical buzz vs. tingling, corresponding to pulse width of 200 μs and 400 μs, respectively, for four out of eight electrodes tested (Fig 6).

#### Spatial discrimination

**Fig 7:**
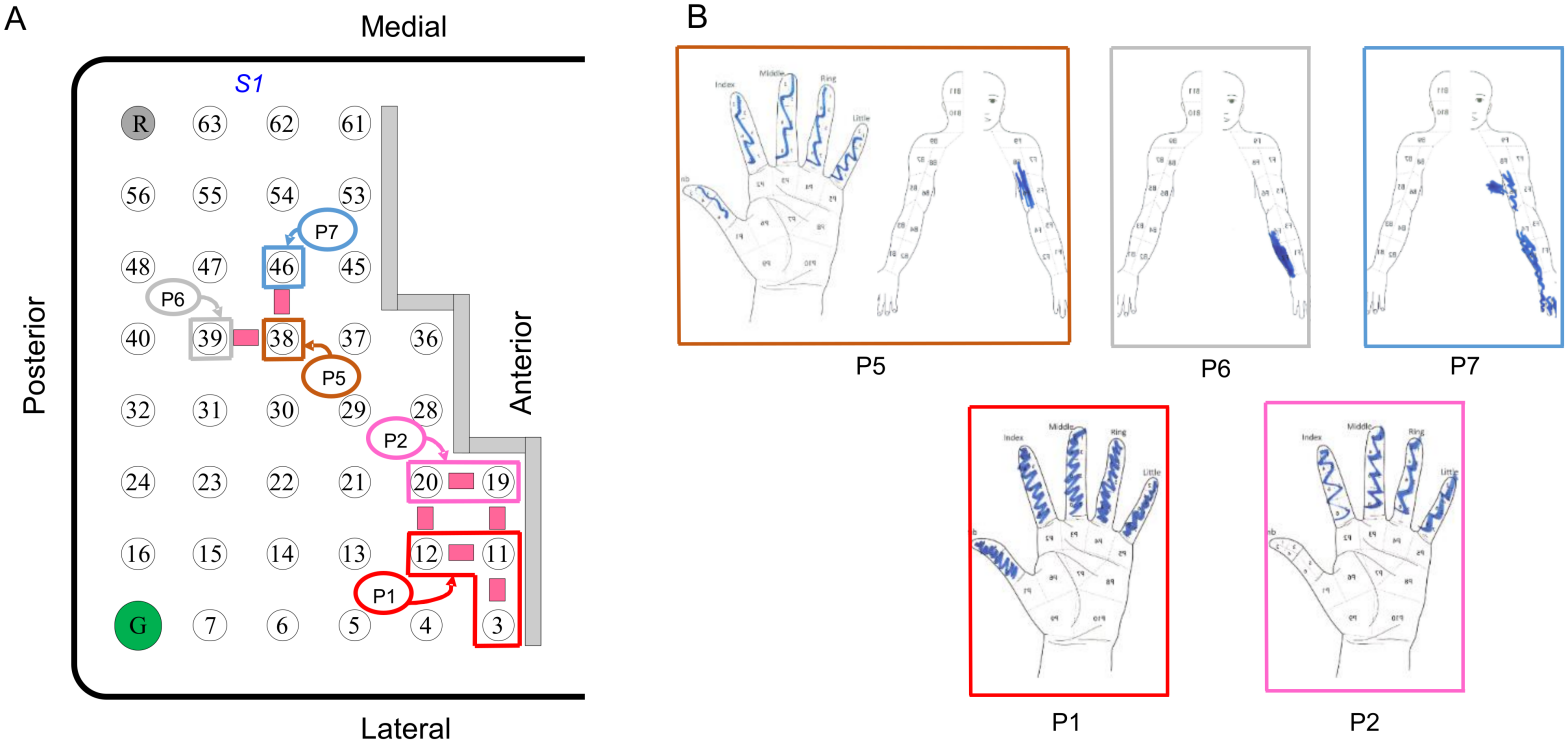
Spatial discrimination for cortical stimulation. ECoG grid showing the pairs of electrodes that were tested for spatial discrimination and their corresponding perceived body parts indicated by colored outlines. The pulse width, pulse frequency, and stimulus train duration were 400 μs, 500 Hz, and 0.5 s, respectively. The pulse amplitude for Electrodes 3, 11, 19, 12, 20, 38, 39, and 46 were 2.5 mA, 1.5 mA, 1.5 mA, 2 mA, 1.5 mA, 2.5 mA, 2 mA, and 2 mA, respectively.

We tested the participant’s ability to tell whether two stimulus trains were delivered at the same or two neighboring electrodes. The participant’s performance for each of the seven pairs of electrodes tested was 50.0% (Electrodes 11 vs. 19), 60.0% (Electrodes 11 vs. 12), 63.3% (Electrodes 3 vs. 11), 73.3% (Electrodes 38 vs. 46), 90.0% (Electrodes 19 vs. 20), 90.0% (Electrodes 38 vs. 39), 93.3% (Electrodes 12 vs. 20). Three of the seven pairs of electrodes (Fig 7) had excellent spatial discrimination (≥ 90% for 30 trials per pair), with others ranging from 50% to 74%.

Primary data presented in this manuscript are available online as S1 File.

## Discussion

The current study found that the somatosensory cortex was intact and that sensations could be evoked by direct cortical surface stimulation three years after complete peripheral nerve injury. There are three specific findings. First, the study quantitatively demonstrated that intensity of perceived sensation increased monotonically with both pulse amplitude and pulse frequency, and the perceived intensity followed a more linear relationship with respect to pulse amplitude than with respect to pulse frequency. Second, we demonstrated that changing pulse width could change the type of sensation. Third, we evoked sensations across a range of locations from the fingers to the upper arm, and for a subset of electrodes, it was possible for the participant to distinguish stimulation applied between neighboring cortical surface electrodes that were only a few millimeters apart. Taken together, the study showed that it was possible to modulate sensation intensity, change sensation type, and operate at a high spatial resolution using cortical surface stimulation. These three capabilities are essential to provide effective somatosensory feedback for neuroprosthetic applications.

We found that the activation threshold for the somatosensory cortex was higher than the motor cortex (Figs 1D and 3). This is consistent with clinical literature about intra-operative mapping of the motor and somatosensory cortices using cortical surface stimulation [24]. It is likely that the motor cortex is more excitable than the somatosensory cortex due to the presence of large pyramidal (Betz) cells and their apical dendritic organization in the pyramidal layer (Layer V) [25]. Cortical mapping also showed that there was spatial clustering of electrodes with the same sensation fields (Fig 4). The sensation field changed gradually from the hand to the arm as the location of the stimulation electrode changed from lateral to medial portion of the ECoG grid, consistent with the somatotopy of the somatosensory cortex [2]. It is worth noting that the participant felt sensation in the intercostal region during stimulation of electrodes over the hand/arm area of the somatosensory cortex (Fig 4, Patterns 7 and 8). It is unclear whether these referred sensations could be related to the intercostal nerve transfer given our limited information regarding the nerve transfer procedure. It will be very interesting to study the effect of nerve transfer surgery on somatosensory cortex, but it is beyond the scope of the current study.

A recent intracortical microelectrode study found that rhesus macaques were able to detect stimulations that were 0.1 s or longer [9], and the recent human intracortical microelectrode study used 1-s stimulus train for stimulation detection [14]. We found that the stimulation duration needed to be at least 0.2 s for our participant to detect the cortical surface stimulation at least 75% of the times. We also found that a time gap of at least 1 s was needed for the participant to detect the existence of two stimulus trains rather than one.

Sensory psychology research has established psychometric curves between probability of stimulation detection and intensity of sensory stimulation [26]. Intracortical microstimulation studies in non-human primates showed that probability of detection of cortical stimulation had a sigmoidal relationship with stimulation pulse amplitude [9]. However, relationship between perceived intensity and pulse amplitude or pulse frequency for cortical surface stimulation has not been studied quantitatively, particularly in humans. Johnson et al. studied able-bodied individuals implanted with standard clinical ECoG grids for seizure monitoring, and they showed that two participants were able to correctly discriminate higher or lower intensity stimulations [16]. We found that the perceived intensity, as reported by the participant, had a monotonic relationship with both the pulse amplitude and pulse frequency. Furthermore, perceived intensity was less variable given the same pulse amplitude than given the same pulse frequency. Perceived intensity also followed a more linear relationship with respect to pulse amplitude than with respective to pulse frequency. Additionally, an increase in pulse amplitude (up to 1 mA above the detection amplitude) did not enlarge the sensation field. This is an important finding because it means sensation intensity can be modulated independently of sensation field. As a hypothetical example, we can potentially provide feedback about the increasing contact force between an object and the hand by increasing pulse amplitude without mistakenly indicating that the contact area is also enlarging. The current study also found that, when two identical stimulus trains were applied sequentially, the perceived intensity of the second stimulus train was lower than that of the first. This may be related to neural adaptation to electrical stimulation, and it needs to be accounted for by stimulation protocols that aim to provide continuous real-time sensory feedback using cortical surface stimulation.

We found the capability to change sensation type by changing pulse width. Johnson et al. have reported that qualitative experience of stimulation was the same for both participants with variation to the pulse amplitude and frequency while keeping the pulse width constant [16]. Our study found that increasing pulse width and decreasing pulse amplitude with constant pulse frequency could change the sensation type in a controlled fashion. The pulse amplitude used with 400-μs pulse was 1/3 to 1/2 of the pulse amplitude used with 200-μs pulse. Hence, the electrical charge exchanged per second remained constant or decreased when pulse width increased from 200 μs to 400 μs, suggesting that pulse width rather than electrical charge had the predominant effect on sensation type. While we were able to change sensation type, our participant reported sensations that were typically less natural, such as tingling and electrical buzz at the hand and arm areas. This is different from the Johnson et al. study [16] and our experience with clinical cortical mapping in neurosurgical patients, where individuals reported complex sensations. Furthermore, the participant of the recent intracortical microstimulation study [14], an individual with spinal cord injury, reported more naturalistic characteristics of evoked sensation. It seems that multiple factors, including both an individual’s baseline neurological condition, particularly their somatosensory function, and stimulation techniques could contribute to naturalness of evoked sensation. This is of both scientific and clinical significance and worth further investigation.

Our investigation of the relationship between pulse width and sensation type is limited in several aspects. First, the sensation types identified in the current study were empirically defined based on the participant’s report. Electrical buzz was reported to be more artificial and closer to the sensation the participant felt when his spinal cord stimulator was on, while tingling was more natural. A more objective and systematic approach to define and characterize the quality of the perceived sensation is needed. Second, only two pulse widths were tested for a limited number of electrodes due to time limitations. Future studies should systematically test a wider range of pulse widths, similar to how we characterized the sensation intensity as a function of pulse amplitude and frequency (Fig 5). Third, pulse width testing was performed using stimulation amplitudes close to the detection threshold. While this approach allowed us to stay below the stimulation safety limit, it likely also generated more variable sensations than higher amplitude stimulation, making it more difficult to study various properties of the elicited sensation. Finally, while we only tested pulse width, multiple stimulation parameters, including pulse width, pulse amplitude, pulse frequency, their potentially complex interaction, and their temporal patterns, i.e., whether they are constant or varying throughout a stimulation train, will likely influence the characteristics of elicited sensation.

Finally, we were able to provide sensory feedback to different body parts at a relatively high spatial resolution across a broad cortical area, the hand and arm area of the somatosensory cortex. Our participant was able to accurately discriminate (≥90%) among three of the seven electrode pairs tested. Since performance varied significantly, we examined two factors that might have affected the spatial discrimination, pulse amplitude and sensation field. First, difference in pulse amplitude between the reference and testing stimulus trains did not seem to affect the performance in spatial discrimination. For example, for electrode pairs (12 vs. 20, 11 vs. 12) where the pulse amplitude of the testing stimulus train was higher than that of the reference stimulus train, the performance was excellent (93.3%) for 12 vs. 20 but poor (60%) for 11 vs. 12. Second, sensation fields of the electrodes pairs–whether they are the same or different–also did not seem to affect the participant’s spatial discrimination performance (11 vs. 12, same sensation fields, 60% accuracy; 19 vs. 20, same sensation fields, 90% accuracy).

The current study has several limitations worth noting. It was conducted in one participant with brachial plexus injury and limited to 28 days. Future studies need to confirm these findings and should evaluate long-term safety of cortical surface stimulation in humans [27]. With limited time and concerns for the theoretical risk of provoking a seizure we limited the number of trials performed, especially compared to studies performed in animal models, and we limited the number of stimulation parameters tested. However, compared to studies conducted in individuals undergoing clinical brain mapping, the current study allowed us to use a custom-designed high-density ECoG grid covering the cortical area of our interest and to work with the participant throughout the 28-day period. We did not attempt real-time closed-loop brain-computer interface application using simultaneous cortical surface recording and stimulation and believe this should be tested in future studies [11, 17].

## Supporting information

S1 File Primary data presented in this manuscript. In compliance with PLOS ONE open data policy, Shared_Data_Cortical_Surface_Stimulation.zip is available for download. This zipped folder contain raw cortical stimulation data (Excel files) that were used to generate the figures and tables in this manuscript.
(ZIP)
